# Sugar metabolism, redox balance and oxidative stress response in the respiratory yeast *Kluyveromyces lactis*

**DOI:** 10.1186/1475-2859-8-46

**Published:** 2009-08-30

**Authors:** M Isabel González-Siso, Ana García-Leiro, Nuria Tarrío, M Esperanza Cerdán

**Affiliations:** 1 Department of Molecular and Cell Biology, University of A Coruña, Campus da Zapateira s/n, 15071- A Coruña, Spain

## Abstract

A lot of studies have been carried out on *Saccharomyces cerevisiae*, an yeast with a predominant fermentative metabolism under aerobic conditions, which allows exploring the complex response induced by oxidative stress. *S. cerevisiae *is considered a eukaryote model for these studies. We propose *Kluyveromyces lactis *as a good alternative model to analyse variants in the oxidative stress response, since the respiratory metabolism in this yeast is predominant under aerobic conditions and it shows other important differences with *S. cerevisiae *in catabolic repression and carbohydrate utilization. The knowledge of oxidative stress response in *K. lactis *is still a developing field. In this article, we summarize the state of the art derived from experimental approaches and we provide a global vision on the characteristics of the putative *K. lactis *components of the oxidative stress response pathway, inferred from their sequence homology with the *S. cerevisiae *counterparts. Since *K. lactis *is also a well-established alternative host for industrial production of native enzymes and heterologous proteins, relevant differences in the oxidative stress response pathway and their potential in biotechnological uses of this yeast are also reviewed.

## Review

### The connections between sugar metabolism, redox balance and oxidative stress

A lot of studies have been carried out on *Saccharomyces cerevisiae*, an yeast with a predominant fermentative metabolism under aerobic conditions [1], which allows exploring the complex response induced by oxidative stress. Recent reviews of different aspects of the oxidative stress response in *S. cerevisiae *have been published but the information about these complex regulatory networks in other yeasts is more limited [2-5]. *Kluyveromyces lactis *is a good model to analyse alternative variants in the oxidative stress response, since the respiratory metabolism in this yeast is predominant under aerobic conditions [6].

A comparison of the transcriptomes of *S. cerevisiae *and *K. lactis*, growing in complete medium with glucose, using heterologous DNA arrays [7], revealed that the transcription of functional groups of genes related to housekeeping functions, such as mitosis, transcription or cell wall biogenesis, is highly correlated in both yeasts. However, large differences between groups of genes related to carbohydrate metabolism, respiratory functions and oxidative stress response have been found.

Several connections between the alternative use of different metabolic pathways and oxidative stress have also been found. The way that sugar oxidation re-routing, carried out by different metabolic pathways, may influence the oxidative stress response is documented both in *S. cerevisiae *[8,9] and *K. lactis *[10]. The *K. lactis rag2 *strain, a mutant lacking the glycolytic enzyme phosphoglucose isomerase, grows in glucose, metabolising the sugar through the Pentose Phosphate Pathway (PPP) but this growth is avoided in the presence of Antimicyn A due to blockade of the mitochondrial respiratory chain after ubiquinone [11]. In the *rag2 *mutant, the preponderance of the use of PPP over glycolysis causes an increase in respiration that restores NADP^+ ^levels and allows the flow through PPP to continue [10]. Growth of the *rag2 *strain PM5-2D in fructose is possible through glycolysis and it is not blocked by Antimicyn A [12]. A moderate increase in mRNAs transcribed from several genes involved in the defence against oxidative stress was observed [7] when comparing the transcriptome of the *rag2 *mutant strain growing in glucose (through PPP) vs. fructose (through glycolysis). This confirms that the use of alternative metabolic pathways in the catabolism of sugars influences the oxidative stress response in *K. lactis*.

It is also possible to find counterpart connections between oxidative stress and the alternative use of metabolic pathways. Hence, the onset of an oxidative stress response may open previously-blocked metabolic pathways. In *S. cerevisiae*, a mutant lacking phosphoglucose isomerase, *pgi1*, does not grow on glucose because the PPP is not fully operative. Growth on glucose of the *pgi1 *mutant is achieved by adding oxidizing agents such as hydrogen peroxide (H_2_O_2_) or menadione, thereby causing oxidative stress to yeast cells [13]. Since the oxidative stress response of *S. cerevisiae *includes up-regulation of genes coding for enzymes that use NADPH as a cofactor, in order to keep reduced glutathione and thioredoxin levels [14], NADPH-dependent stress mechanisms are a metabolic supply of oxidized NADP^+ ^[15]. In these conditions, the mutant yeast cells adapt their metabolism to obtain the extra NADPH needed during the stress response by redirecting carbohydrate fluxes to the PPP to the detriment of glycolysis [16]. A recent study [17] has shown that the ability to redirect metabolic fluxes from glycolysis to the PPP in response to oxidative stress in order to obtain reduced coenzymes is conserved between yeasts and animals, outlining their importance in the adaptation to oxidative stress.

Redox signalling might also control metabolic fluxes through enzymatic regulation. Recently, it has been hypothesized that *Kl*AdhI (homotetrameric cytosolic alcohol dehydrogenase I) might represent an important target in redox signalling in *K. lactis *cells. *In vitro*, there is a *Kl*AdhI wild-type in two reversible forms: reduced (active) and oxidized (inactive) with the Cys278 residues of each tetramer linked by disulphide bonds. Oxidized glutathione is one of the agents that inactivate the enzyme. The redox state of *Kl*AdhI could be a mechanism for modulating the enzyme activity directly and the glucose flux through glycolysis or PPP indirectly [18].

In *S. cerevisiae*, it has been described that glucose limitation (caloric restriction) promotes a decrease in reactive oxygen species (ROS) formation and an increase in longevity that does not occur in *K. lactis *[19]. The authors explain this difference by the fact that whereas *S. cerevisiae *shows catabolic repression of respiration (alleviated by low glucose levels), *K lactis *does not. These data reinforce the idea of different interrelationships between glucose metabolism and oxidative stress in respiratory or fermentative yeasts.

**Figure 1:**
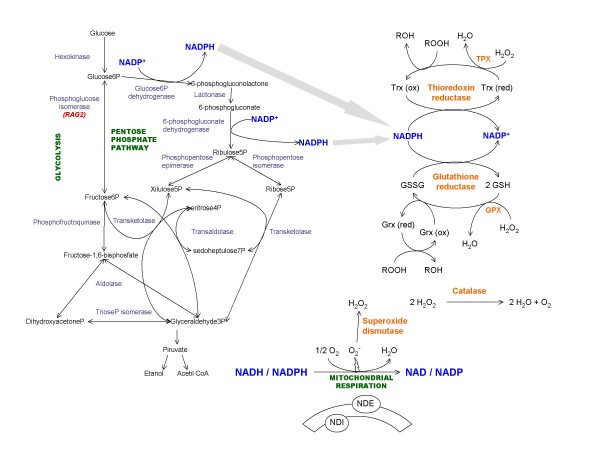
**Interrelationship between the oxidative stress response, sugar metabolism and redox balance in *Kluyveromyces lactis***.

Taking into account the above-described interconnections between metabolic fluxes and oxidative stress (Figure 1), it is possible to envisage the *K. lactis *model as a very fruitful system to study regulatory mechanisms affecting the oxidative stress response of a respiratory yeast and to compare them to the previously reported features for the fermentative yeast *S. cerevisiae*. Although data are still limited in *K. lactis*, we review similarities and differences already reported, or deduced from genomic comparative analysis and affecting important aspects of the oxidative stress response in yeasts. We consider the production of ROS, enzymatic reactions producing ROS detoxification, repair of oxidative damage caused in proteins and lipids and the implications of transcriptional regulators in these processes. Finally, we review related biotechnological applications, which can be exploited in a near future using *K. lactis *systems.

### Alternative dehydrogenases, respiratory chain and generation of ROS in *K. lactis *cells

ROS are a group of molecules derived from molecular oxygen, such as peroxides, including H_2_O_2_, alkylhydroperoxides, the hydroxyl radical and the superoxide anion. ROS have toxic effects but also regulatory functions. Oxidation and reduction of thiol proteins are thought to be the major mechanisms by which ROS integrate into cellular signal transduction pathways [20]. An excess of ROS results in oxidative stress and may eventually cause cell death. The sources of ROS are either exogenous (heavy metal ions, γ-radiation, UV light) or endogenous. The leakage of electrons from the mitochondrial respiratory chain has been described as the major source of endogenous ROS under physiological conditions, and the components of the initial and middle segments of the chain are the most active producers in this regard [21].

The respiratory chains of the yeasts *S. cerevisiae *and *K. lactis *are characterized by the lack of complex I and the presence of three alternative NADH dehydrogenases located at the mitochondrial inner membrane. These are rotenone-insensitive and single-polypeptide enzymes that transfer the electrons to ubiquinone without proton pumping. The NADH generated in the mitochondrial matrix is oxidised by the internal alternative dehydrogenase Ndi1, while the external enzymes, Nde1 and Nde2, oxidise cytosolic NADH directly [22]. Unlike *S. cerevisiae*, the external alternative dehydrogenases of *K. lactis *also oxidises cytosolic NADPH [23,24].

In *S. cerevisiae*, ROS production by the electron transport chain was initially associated to complex III and to external alternative dehydrogenase [25,26]. Later, it was also associated to the internal alternative dehydrogenase [27]. Li et al. [27] described that the disruption of Ndi1 and Nde1 in *S. cerevisiae *decreases ROS production and prolongs life span. However, in *K. lactis*, the null mutants either in the external [24] or internal alternative dehydrogenases do not show decreased levels of ROS when compared to the wild-type strain (our unpublished data). Although experimental data confirm a similar organization of the dehydrogenases, which allows cytosolic NAD(P)H or mitochondrial NADH reoxidation by the respiratory chain in *S. cerevisiae *and *K. lactis*, ROS production in the two yeasts differs in mutant defective from the homologous dehydrogenases.

To understand this difference between structural and functional data, it is interesting to note that ROS production is not only determined by the organization of the components of the respiratory chain, but also by the relative flow of NAD(P)H re-oxidation that is achieved through the mitochondrial chain or by other systems. Several examples of these mechanisms which produce differences in relative metabolic fluxes are found. The *rag2 *mutant from *K. lactis *is more resistant to oxidative stress and produces more ROS than the wild-type strain in aerobic cultures with glucose as carbon source [28]. Transcription levels of *KlSOD1, KlCTA1 *and *KlCTT1*, necessary for ROS detoxification, are higher in the *K. lactis rag2 *mutant than in the wild-type strain, when they are growing in glucose, even in the absence of an exogenously induced oxidative stress [29]. The *K. lactis *mutant *rag2 *has to re-route all the glucose from glycolysis through the oxidative branch of the PPP and therefore, the increase in ROS production could be attributed to the higher activity of the mitochondrial external alternative dehydrogenases to oxidise the surplus of NADPH [10,30]. The double null mutant in phosphoglucose isomerase and Nde1, the most important of the two external enzymes for NADPH oxidation, does not grow on glucose [28] and then a putative decrease in ROS levels in such a double mutant cannot be experimentally verified. In the *rag2 *mutant grown in glucose, the transcription of *KlNDE1 *decreases by the addition of 0.4 mM H_2_O_2 _to the medium [23], when NADPH-consuming mechanisms of defence to oxidative stress are increased [31]. This transcriptional regulation also affects *KlNDE2*, the gene of the second *K. lactis *external alternative dehydrogenase using NADPH [24], and other genes related to active respiration, as revealed by the data obtained through the use of DNA arrays [29]. In summary, NADPH reoxidation through the respiratory chain decreases when the NADPH-dependent oxidative stress defence reactions are up-regulated.

As explained above, the blockade of electron flow by disruption of alternative dehydrogenases in *K. lactis *and *S. cerevisiae *has different consequences on ROS production. However, when the electron flow is disrupted downstream in the electron chain by the inhibitor of the cytochrome *bc1 *complex antimycin A, similar results are obtained in the two yeasts. In *S. cerevisiae*, an increase in mitochondrial H_2_O_2 _production is observed [25]. Although there are no direct data available on the influence of antimycin A in ROS production in *K. lactis*, we have observed (unpublished data) that antimycin A increases tolerance to peroxide-mediated oxidative stress both in *S. cerevisiae *and *K. lactis*. This might result from the up-regulation of antioxidant defences pointed out by increased ROS levels.

Eukaryotic cells have developed several defence systems against ROS. We summarize below the information available at present on some of these systems in *K. lactis*.

### Genes encoding enzymes for ROS detoxification and glutathione synthesis are conserved in *K. lactis *and *S. cerevisiae*

**Figure 2:**
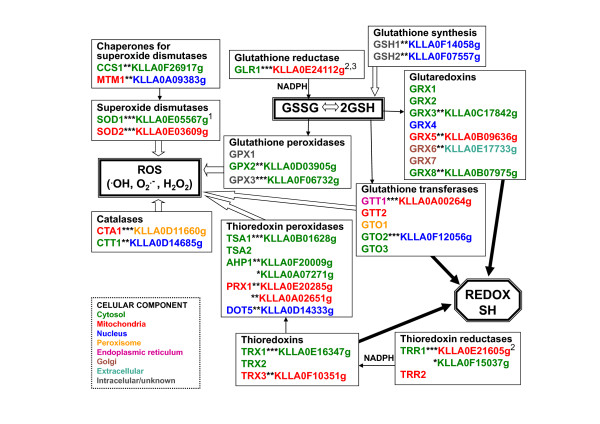
**Putative oxidative stress response *Kluyveromyces lactis *sequences and their *Saccharomyces cerevisiae *counterparts**. Similarity of the proteins (*** = highly similar ** = similar * = weakly similar). For *K. lactis *proteins, the cellular location indicated corresponds to the highest probability estimated by WoLF PSORT [36], and for *S. cerevisiae *proteins to the first location mentioned in SGD (Saccharomyces Genome Database); ^N ^= reference for cloning/expression of the *K. lactis *gene: ^1 ^[37], ^2 ^[31], ^3 ^[28]. See also Additional File 1.

The analysis of the complete sequence of the *K. lactis *genome, available through Génolevures [32], allows finding putative orthologs to *S. cerevisiae *genes which are related to ROS detoxification and glutathione synthesis. These include genes coding for supexoxide dismutases and their chaperones, catalases and peroxidases, glutathione and thioredoxin systems. The results summarized in Figure 2 (see Additional File 1) reveal that, in general, these genes are well-conserved in the two yeasts. Specific comments and other experimental information on particular *K. lactis *genes are detailed in the following sections. The systematic Génolevures nomenclature is used throughout the paper for the *K. lactis *sequences, synonyms are given in brackets.

#### Superoxide dismutases

Superoxide dismutases (SODs) catalyze the breakdown of the superoxide radical to an oxygen molecule (O_2_) and H_2_O_2_. Two SODs are present in *S. cerevisiae *and other yeasts, a Cu-Zn containing form in the cytosol (Sod1) and an Mn-containing form in the mitochondrion (Sod2) [33]. The active form of Sod1 is a homodimer in *S. cerevisiae*. Two conserved Cys of each monomer are joined together in a disulphide bond and this bond is critical for the enzymatic activity. The specific copper chaperone Ccs1 delivers the copper ion to Sod1 and also facilitates the formation of intramolecular disulphide bond [34]. Sod2 is a homotetramer in *S. cerevisiae*. The specific manganese chaperone Mtm1 delivers the metal ion to Sod2, in the mitochondrial matrix [35].

The proteins Sod1, Sod2 and their chaperones have orthologs in *K. lactis*: KLLA0E05567g (KLLA0E05522g) is highly similar to *S. cerevisiae *Sod1, KLLA0E03609g (KLLA0E03509g) is highly similar to *S. cerevisiae *Sod2; KLLA0F26917g is similar to *S. cerevisiae *Ccs1 and KLLA0A09383g is similar to *S. cerevisiae *Mtm1. The alignment between orthologs shows that the residues important for the activity and interaction with metallic cofactors are conserved between the corresponding proteins of the two yeasts. The sub-cellular localization predicted by WoLF PSORT [36] is mainly cytosolic for *Kl*Sod1 and mitochondrial for *Kl*Sod2 (Figure 2 and Additional File 1), as reported for their *S. cerevisiae *orthologs.

In spite of the pairwise similarity in the proteins of this group, several differences between these enzymes in *K. lactis *and *S. cerevisiae *have been reported. Thus, *KlCCS1 *overexpression has not increased *Kl*Sod1 activity and a different mechanism for cation handling in *Kl*Sod1 has been proposed, also considering the absence of two Pro residues near the C-terminus [37]. Other reported differences are related to transcriptional regulation. In *S. cerevisiae *mRNA levels of several genes of the stress response increase during hypoxia [38-40]. The response to hypoxia in this group of genes in *K. lactis *is low [29]. *S. cerevisiae SOD1 *shows a decreased expression after a shift to anaerobiosis for up to 4 h, and then it increases to levels higher than those in normoxia [40]. On the contrary, *KlSOD1 *does not show increased expression after 6 h of a shift from aerobiosis to hypoxia [29].

#### Catalases

Catalase breaks down H_2_O_2 _into O_2 _and H_2_O molecules using the redox properties of a protein-heme complex. In *S. cerevisiae*, catalase is coded by two genes, *CTA1 *and *CTT1*, corresponding to two isoforms with different subcellular locations, peroxisomal-mitochondrial matrices and cytosol, respectively [38,41]. *S. cerevisiae *Cta1 is a homotetramer with a heme group and a NADP(H) one, cofactor binding sites per subunit [42]. *K. lactis *orthologs are KLLA0D11660g, highly similar to *S. cerevisiae *Cta1, and KLLA0D14685g, similar to *S. cerevisiae *Ctt1 (Figure 2 and Additional File 1). Both *K. lactis *catalases show the typical heme-ligand signature as determined by the Motif Scan programme [43].

In *S. cerevisiae*, the transcription of *CTA1 *and *CTT1 *is induced under aerobic conditions and *CTT1 *is also induced under several stresses [44,45]. This transcriptional regulation is not observed in *K. lactis *[29]. Although catalase activity is increased by addition of peroxides and after an aerobiosis-hypoxia shift, this increase might be attributed to post-transcriptional mechanisms [28].

In *S. cerevisiae*, the effects of catalase and glutathione in defence against H_2_O_2 _overlap. Thus, the absence of catalases enhances the hypersensibility to oxidants of a strain unable to synthesize glutathione [46] and mutants in catalase show decreased resistance to oxidative stress [47,48]. A similar interdependence of both systems is also detected in the *rag2 *mutant of *K. lactis *which has increased resistance to oxidative stress compared to the wild type. When catalase is inhibited by 3-aminotriazole, the tolerance to peroxide-mediated oxidative stress is reduced, and this effect is more evident when the gene encoding glutathione reductase is also deleted [28].

Although H_2_O_2 _is a strong oxidizing agent, most of its reactions have high activation energy and are slow; H_2_O_2 _reacts directly with a few chemical groups including thiols [20]. In catalase deficient cells, if the redox buffering capacity of glutathione is also decreased, high sensitivity to peroxides will be produced, mainly because they react with thiols from proteins, therefore altering their functions.

#### Peroxidases

Peroxidases reduce inorganic and organic peroxides into the corresponding alcohols using active site cysteine thiols. Two classes of peroxidases are distinguished according to the electron donor for the thiols, glutathione peroxidases (GPXs) and thioredoxin peroxidases or peroxiredoxins (TPXs); although GPXs use sometimes thioredoxin and TPXs use glutathione as electron donors [49,50]. GPXs are classified as soluble and membrane-associated, these latter are also called Phospholipid hydroperoxide GPXs, and they reduce soluble hydroperoxides and also/or phospholipid hydroperoxides from membranes, respectively.

##### Glutathione peroxidases

The three GPXs described in *S. cerevisiae*, Gpx1, Gpx2 and Gpx3 (Hyr1), are phospholipid hydroperoxide GPXs [51].*S. cerevisiae *Gpx1 and Gpx2 are induced by glucose starvation and Gpx3 senses intracellular hydroperoxide levels to transduce a redox signal to the transcription factor Yap1p. The cellular locations of Gpx1 and Gpx3 are unknown, Gpx2 is found in cytosol and nucleus [51-54]. In Génolevures the *K. lactis *protein showing the highest identity with *S. cerevisiae *GPXs is KLLA0F06732g that shows 80% identities with Gpx3 (Hyr1), and also 75% identities with Gpx2 and 59% identities with Gpx1. The sequence KLLA0D03905g, annotated for Gpx2, shows 57% identities with *S. cerevisiae *Gpx2 and with Gpx3 (Hyr1) and 46% with Gpx1 (Figure 2 and Additional File 1).

Besides the structural similarities between the *K. lactis *and *S. cerevisiae *genes, the transcription of orthologs is induced by oxidative stress in both yeasts. The transcription of *S. cerevisiae GPX2 *[55] and the two *K. lactis *sequences showing similarity, mainly KLLA0F06732g, are strongly induced by H_2_O_2 _[29].

##### Thioredoxin peroxidases

There are five different TPXs in *S. cerevisiae*, found at different cellular compartments: Tsa1, Tsa2 and Ahp1 are cytosolic, Prx1 is mitochondrial and Dot5 is nuclear [56]. The *K. lactis *sequences annotated for the corresponding genes in the genome database are as follows: KLLA0B01628g for Tsa1 (this protein shows also high similarity to *S. cerevisiae *Tsa2, 83% identities), KLLA0A07271g and KLLA0F20009g for Ahp1, KLLA0E20285g (KLLA0E20383g) and KLLA0A02651g for Prx1, KLLA0D14333g for Dot5 (Figure 2 and Additional File 1). *TSA1 *transcription is strongly induced by H_2_O_2 _in *K. lactis *[29]. It is remarkable that, although gene redundancy is generally lower in *K. lactis *than in *S. cerevisiae *[32], the opposite is true for this particular group of genes. The cellular locations predicted with the highest probability for the *K. lactis *proteins by WoLF PSORT [36] coincide with the locations of the *S. cerevisiae *counterparts (Figure 2 and Additional File 1).

This group of enzymes are proposed to be *moonlighting *proteins, at least in *S. cerevisiae *[2]. That is, they show several functions and are able to participate in unrelated biological processes [57]. For example, besides their peroxidase activity, Tsa1 shows chaperone activity and Dot5 takes part in the disruption of telomeric silencing [58,59]. It has been shown that *moonlighting *activities are not necessarily conserved among yeast species [57] and, to our knowledge, there are no functional studies on these proteins that allow assigning or discarding alternative functions for these proteins in *K. lactis*.

#### Glutathione biosynthesis

The two *K. lactis *sequences annotated as genes for the biosynthesis of glutathione are KLLA0F14058g (*KlGSH1*), encoding a putative Gamma glutamylcysteine synthetase that catalyzes the first step in glutathione biosynthesis and KLLA0F07557g (*KlGSH2*), encoding a putative Glutathione synthetase that catalyzes the ATP-dependent synthesis of glutathione from gamma-glutamylcysteine and glycine (Figure 2 and Additional File 1).

In *S. cerevisiae, GSH1 *and *GSH2 *expression is induced by oxidants, such as H_2_O_2_, and by heat shock, both types of regulation mediated by the Yap1 transcription factor [60-63]. In the yeast *Pichia pastoris*, the genes of glutathione synthesis are also regulated by the *Pp*Yap1 transcription factor [64]. The expression of the *K. lactis *orthologs, on the contrary, is not induced by H_2_O_2_, although *KlGSH1 *is one of the few oxidative stress response genes whose transcription is induced after a shift to hypoxia [29]. In this regard,*K. lactis *appears different to *S. cerevisiae *and also to other non-*Saccharomyces *yeasts.

### Similarities and differences between glutathione and thioredoxin systems from *S. cerevisiae *and *K. lactis*

The arrangement of genes from the thioredoxin and glutaredoxin systems, responsible for the repair of oxidative protein damage, shows several differences in *K. lactis *and *S. cerevisiae *as described here below.

#### The thioredoxin system

The thioredoxin system is made up of thioredoxin (TRX), thioredoxin reductase (TRR) and NADPH. TRR uses a dithiol-disulphide active-site to transfer, via the cofactor FAD, reducing equivalents from NADPH to TRXs, which are thiol oxidoreductases with two cysteines at the active site. This system, by reducing disulphide bonds, participates in the regulation of the activity of enzymes such as ribonucleotide reductase but also in protein folding and in redox signalling, this latter including transcriptional regulation of gene expression. Thus, Yap1, the transcriptional regulator of the yeast response to peroxides, is activated through oxidation mediated by peroxides and deactivated through reduction mediated by thioredoxin.*S. cerevisiae *contains two separate thioredoxin systems. The cytosolic system is made up of two TRXs (Trx1, Trx2) and one TRR (Trr1) and the mitochondrial one consists of one TRX (Trx3) and one TRR (Trr2) [65,66].

In the *K. lactis *genome database, there is one sequence annotated as a putative gene for *TRX1*, KLLA0E16347g (KLLA0E16401g), and another for *TRX3*, KLLA0F10351g, but there is no ortholog for *TRX2 *(Figure 2 and Additional File 1). The thioredoxin-active site-related signature (APWCGHCK or APWCGYCQ) was also found in the *K. lactis *protein disulphide isomerases *Kl*Pdi1 and *Kl*Mpd1 [67].

Two sequences in Génolevures are annotated as *TRR1 *genes KLLA0E21605g (KLLA0E21692g) and KLLA0F15037g. The first is highly similar to *S. cerevisiae *Trr1 and the second is only weakly similar. KLLA0E21605g is also highly similar to *S. cerevisiae *Trr2 (78% identities) and there is no other *K. lactis *sequence annotated as a putative gene for *TRR2 *(Figure 2 and Additional File 1). Between KLLA0E21605g and KLLA0F15037g, no significant alignment of proteins is produced. There are only 23% identities and the overlap includes 180 residues although the lengths of the proteins involve 350 and 298 residues respectively. The protein encoded by KLLA0E21605g shows a mitochondrial export signal of 29 residues, as predicted by MitoProt II [68] with a probability of 0.99; KLLA0F15037g is predicted to be cytosolic with WoLF PSORT [36].

**Figure 3:**
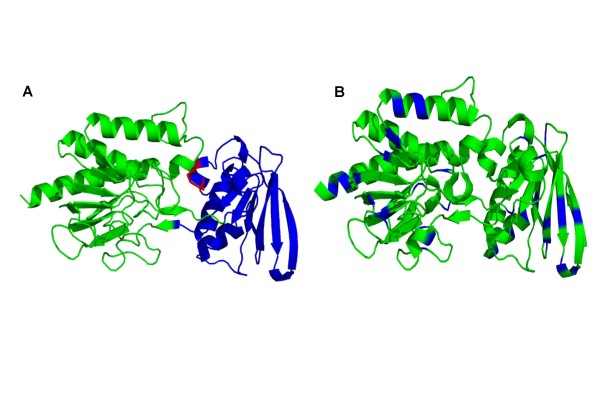
**3D-model of *Kluyveromyces lactis *thioredoxin reductase performed with PyMOL Molecular Viewer  (A) Domains are marked in colours**. Green: FAD binding domain (residues 32-156 and 281-349). Blue: NADPH binding domain. The two Cys that make up the active site are marked in red. (B) Residues different from *S. cerevisiae *cytosolic thioredoxin reductase are marked in blue, these differences do not affect significantly the overall structure of the protein.

Among the genes of the *K. lactis *thioredoxin system, only KLLA0E21605g has been studied and its TRR activity has been proven [31,69]. TRR enzymatic activity has been detected both in the cytosolic and mitochondrial fractions of *K. lactis *cells (our unpublished data). However, since the function of KLLA0F15037g remains to be proven hitherto, it is not possible to state whether mitochondrial and cytosolic TRRs in *K. lactis *are encoded by a single gene (KLLA0E21605g) or by two genes. TRRs occur in two forms, a high molecular weight enzyme such as those of mammals, the malaria parasite *Plasmodium falciparum *and some worms, and a low molecular weight form that is present in bacteria, fungi, plants and some protozoan parasites [70]. The protein encoded by *KlTRR1 *belongs to the group of low molecular weight TRRs (homodimers, about 35 kDa/subunit) and shows their characteristic features [31]. Mammalian TRRs have an additional C-terminal domain containing a selenocysteine residue at the penultimate position [71], which is absent in *Kl*Trr1p [31]. Recently, the *S. cerevisiae *TRR structure has been solved and it shares a very similar overall structure to *Escherichia coli *TRR. However, fine comparisons indicate differences at the TRX recognition sites [72]. The predicted 3D structure of *Kl*Trr1 is similar to the *S. cerevisiae *homologue (Figure 3).

The *S. cerevisiae *genes *TRR1/TRR2 *are Yap-1 targets induced by H_2_O_2 _[73] and the same is true for *KlTRR1*. The addition of peroxides (H_2_O_2 _and tBOOH) increases transcription of *KlTRR1 *and also TRR enzymatic activity [28,29,31]. Besides, a consensus for Yap1p binding (ATGAATCAG at position -231 to -223) is functional in the *KlTRR1 *promoter, as demonstrated by the technique of promoter-*lacZ *fusions and beta-galactosidase activity measurements [31].

#### The glutathione/glutaredoxin system

Besides glutathione and glutaredoxins (GRXs), this system is made up of glutathione reductase (GLR) and NADPH. GRXs are small heat-stable thiol oxidoreductases using the tripeptide glutathione (gamma-glutamil-cysteinyl-glycine) as hydrogen donor. Reduced glutathione (GSH) is regenerated from glutathione disulphide (GSSG) by GLR that uses NADPH as a reducing source and FAD as a coenzyme.

The *S. cerevisiae *genome includes eight GRXs identified hitherto, three dithiol GRXs (Grx1, Grx2 and Grx8) with the CPY/FC motif at the active site, and five monothiol GRXs (Grx3, Grx4, Grx5, Grx6 and Grx7) with the CGFS motif at the active site. Grx1 and Grx2 are located at the cytosol, a fraction of Grx2 is also present at the mitochondria, Grx3 location is at the cytosol-nucleus, Grx4 is at the nucleus and Grx5 localizes at the mitochondrial matrix. Grx1 protects cells against hydroperoxides and superoxide-radicals, Grx2 also exhibits a glutathione peroxidase activity, Grx3 and Grx4 sense the iron status of the yeast cells and regulate the nuclear localization of the Aft1 transcription factor, and Grx5 participates in the late stages of the biosynthesis of Fe/S clusters. Grx6 and Grx7 are located at the *cis-*Golgi and associated with the early secretory pathway. Finally, Grx8, which is localized at the cytosol, has several novel structural and mechanistic features [74-80]. GLR in *S. cerevisiae *is coded by a single gene, *GLR1*, which gives rise to a protein with a double location (cytosol and mitochondria) due to the alternative use of two translation initiation sites [81].

In the *K. lactis *genome database, only four sequences are annotated as genes for GRXs: KLLA0C17842g for *GRX3*, KLLA0B09636g for *GRX5*, KLLA0E17733g for *GRX6 *and KLLA0B07975g for *GRX8*. The sequence KLLA0E24069g is annotated for *GLR1*. The predicted subcellular location of these proteins with WoLF PSORT [36] is at the cytosol-nucleus for *Kl*Grx3, mitochondria for *Kl*Grx5 and *Kl*Glr1, extracellular for *Kl*Glr6 and cytosol for *Kl*Glr8 (Figure 2 and Additional File 1).

*KlGLR1 *(KLLA0E24069g) [69] is the only gene experimentally studied from this system in *K. lactis *so far. Overexpression of the *KlGLR1 *gene in a multicopy plasmid, under the control of its own promoter, causes an 8-fold increase in GLR activity when compared to wild-type levels [31]. Moreover, a null mutant in the *KlGLR1 *gene shows no glutathione reductase activity [28]. Although this result confirms that *KlGLR1 *is the only gene that encodes a functional glutathione reductase in *K. lactis*, GLR activity is present both in mitochondria and cytosol (our unpublished data) suggesting a possible mechanism of sorting to mitochondria.

**Figure 4:**
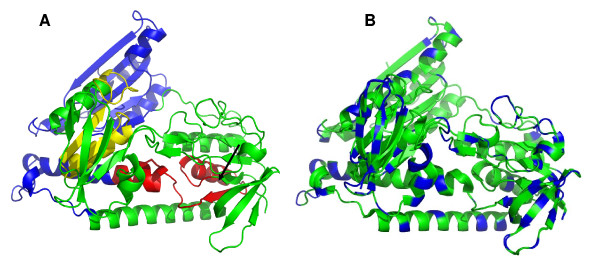
**3D-model of *Kluyveromyces lactis *glutathione reductase performed with PyMOL Molecular Viewer  (A) Domains are marked in colours**. Blue: dimerization domain. Yellow: NADPH binding domain. Red: FAD binding domain. The arrow points out the disulphide bridge that makes up the catalytic domain (B) Residues different from *S. cerevisiae *glutathione reductase are marked in blue, these differences do not affect significantly the overall structure of the protein.

*S. cerevisiae *Glr1 is a dimeric flavo-oxidoreductase whose structure has been solved [82]. The overall structure and the active site are conserved in the *E. coli *and human homologues but differences are found at the interface of the monomers, mainly in the region of the N-terminal domain that contributes to the formation and stabilization of homodimers. The protein encoded by *KlGLR1 *exhibits a similar predicted 3D structure and distribution by binding domains (Figure 4). The N-terminal FAD-binding domain contains a glycine-rich motif GXGXXG/A involved in the binding of the coenzyme and a redox-active disulphide, necessary for electron flow between NADPH and oxidized glutathione via FAD. The central NADPH-binding domain also contains a glycine-rich GXGXXG/A motif but with residues, distinctive for specific interaction with NADP(H). The C-terminal domain makes up the interface between subunits in the dimeric proteins [31].

In *K. lactis*, Glr1 regulation does not respond to peroxide treatment, neither by changes in mRNA transcription of the *KlGLR1 *gene nor by enzymatic activity modulation [28,29,31], whereas *S. cerevisiae *Glr1 is an oxidative stress-defence inducible enzyme and its gene is a Yap1p-target [83,84]. In spite of the lack of induction of *KlGLR1 *by peroxides, the influence of *Kl*Glr1 in oxidative stress resistance is inferred from the fact that the tolerance to H_2_O_2 _of the *rag2 *mutant decreases when the *KlGLR1 *gene is deleted and also because overexpression of *KlGLR1 *increases tolerance to H_2_O_2 _[28].

#### The role of TRR1 and GLR1 from *K. lactis *in NADPH reoxidation

In addition to their participation in oxidative stress defence, TRR and GLR activities also contribute to the reoxidation of the surplus of cytosolic NADPH produced in the PPP in *K. lactis*, although to a lesser extent than other mechanisms recently reviewed [10]. This could be regarded as a functional difference between these enzymes in *K. lactis *and *S. cerevisiae*.

As explained above, the *K. lactis rag2 *mutant growing in glucose (re-routing this sugar through PPP) or fructose (glycolysis) produces different NADPH cytosolic levels. When the *K. lactis rag2 *mutant grows in glucose, there is a transcriptional gene induction of the external alternative dehydrogenases if compared with mRNA levels obtained for the *rag2 *mutant growing in fructose or the wild-type strain growing in glucose. This induction is necessary for rapid NADPH reoxidation by these enzymes and it is lowered after treatment with H_2_O_2_, i.e. after induction of the NADPH-consuming defence mechanisms against oxidative stress, and specifically TRR [23,29]. The comparison of GLR activity in the *K. lactis rag2 *mutant growing on glucose vs. fructose and vs. the wild-type strain shows a small but significant increase [10,28] that is not shown at a transcriptional level [29,31].

To test the relative importance of these enzymatic activities, external mitochondrial dehydrogenases vs. TRR or GLR, for NADPH reoxidation, it was also assayed whether the impaired growth on glucose of the *rag2 *mutant when the respiratory chain was blocked, either by Antimycin A or by hypoxia, could be restored by means of increasing TRR or GLR activity. The result was negative in both assays [28]. The *rag2 *mutant does not grow under hypoxia in spite of the hypoxic increase in *KlTRR1 *expression and enzymatic activity in the *rag2 *mutant, compared to the wild-type strain [29] and it does not grow on glucose with Antimycin A even if *KlTRR1 *expression is induced by peroxide treatment [28]. In a similar way, *KlGLR1 *overexpression does not restore growth on glucose of the *rag2 *mutant when the mitochondrial reoxidation of cytosolic NADPH is blocked by Antimycin A [28].

The role of GLR activity in cytosolic NADPH reoxidation is supported by the remark that the expression of the *KlGLR1 *gene, under its own promoter in an episomal plasmid, completely restores the growth on glucose of the *S. cerevisiae pgi1 *mutant [10]. In *S. cerevisiae *and other organisms, GLR has been reported to regulate the activity of glucose-6-P dehydrogenase by controlling the NADP^+^/NADPH ratio through redox interconversion of glutathione [85]. In fact, the increase of GLR activity in the *K. lactis rag2 *mutant is positively correlated with the glucose-6-phosphate dehydrogenase (G6PDH) activity that produces NADPH and it is also positively regulated by an active respiratory chain [28].

Moreover, to support further the role of GLR in cytosolic NADPH reoxidation in *K. lactis*, the growth on glucose of the double null mutant Δ*Klglr1Δrag2 *is improved in comparison with the *rag2 *strain. This was attributed to the deviation of NADPH from GLR to the mitochondrial dehydrogenases. Thus, more energy is obtained, since the reoxidation of the NADPH from the PPP by mitochondrial external dehydrogenases yields ATP but the reoxidation by GLR does not [28].

#### Glutathione transferases in *K. lactis*

The glutathione transferases (GSTs) function as detoxifiers of electrophilic compounds such as xenobiotics, anticancer drugs, heavy metals or products of oxidative stress by conjugating them to GSH and excreting the GSH-conjugated molecules with improved solubility. Structurally, GSTs belong to the thioredoxin-fold group. In *S. cerevisiae *there are two standard GSTs (Gtt1 and Gtt2), which overlap functionally with Grx1 and Grx2. Gtt1 is associated to the endoplasmic reticulum and Gtt2 is mitochondrial [86,87]. Also, *S. cerevisiae *contains three omega-class GSTs (Gto1, Gto2 and Gto3), which are not active against standard GSTs substrates but are active as thiol oxidoreductases (GRXs). They make up a mixed disulphide between GSH and a N-terminal Cys of the GST molecule. Gto1 is peroxisomal, Gto2 and Gto3 are cytosolic [52,88].

The *K. lactis *genome database contains two sequences annotated for GSTs: KLLA0A00264g is highly similar to *S. cerevisiae *Gtt1 and KLLA0F12056g is similar to *S. cerevisiae *Gto2. KLLA0F12056g also shows 55% identities with *S. cerevisiae *Gto3. The sub-cellular localization predicted by WoLF PSORT [36] is mainly mitochondrial for *Kl*Gtt1 and nuclear for *Kl*Gto2 (Figure 2 and Additional File 1). Since no further information is available on these genes in *K. lactis*, the comparison with the situation in *S. cerevisiae *is waiting for future experimental data reports.

### Transcriptional regulators of the response to oxidative stress in *K. lactis*

#### Comparison of the Yap family of b-ZIP proteins in S. cerevisiae and *K. lactis*

In *S. cerevisiae *the Yap1 transcriptional factor (for yeast AP-1 factor) is the major regulator of the oxidative stress response. It was initially observed that the Δ*yap1 *deletion mutant is hypersensitive to peroxides, H_2_O_2 _and t-BOOH, and also to chemicals which generate superoxide anions. These latter include menadione, plumbagine and methylviologen. Δ*yap1 *is also hypersensitive to cadmium, methylglyoxal and cycloheximide. Yap1 is therefore central to the adaptive response to oxidative stress, regulating not only the response to H_2_O_2_-induced stress, but also that to chemical oxidants (redox cycling chemicals, thiol oxidants and alkylating agents), cadmium and drug stress [89-91].

In *S. cerevisiae*, the Yap family of b-ZIP proteins comprises eight members (Yap1-Yap8) with a significant sequence similarity to the yeast factor Gcn4 at the DNA-binding domain [89]. The Yap family is involved in a variety of stress-related programmes, including the response to DNA damage and oxidative, osmotic, and toxic metal stresses. The members of the Yap family carry out overlapping but distinct biological functions.

*YAP2 *overexpression confers resistance to cadmium, cerulenin and 1,10-phenanthroline, among others [90] and the yap2 null shows decreased resistance to oxidative stress and 5-fluoruracil . Yap4 and Yap6 are the Yap family members which share the greatest similarity at the protein level, showing almost 33% identity between them [91]. *YAP4 *is induced under hyperosmotic stress and regulated by Msn2 in a Hog1-dependent way via the STRE element present in the upstream promoter region [92].

In *S. cerevisiae*, resistance to arsenic is achieved through the activation of the arsenic compounds-resistance (ACR) cluster [93], which is made up of the positive regulator Acr1 (Yap8), the arsenate-reductase Acr2 and the plasma membrane arsenite efflux protein Acr3 [94]. The *YCF1 *(yeast cadmium factor) gene encodes an independent detoxification system that also sequesters arsenic into the vacuole [95,96]. Induction of the expression of *ACR2*, *ACR3 *and also *YCF1 *by the transcription factor Yap8 is essential to arsenic stress response [97]. Besides, Yap1, 2, 4, 5, and 6 have been related to the cellular response to methylmethanesulfonate (MMS), a DNA alkylating agent [98-104].

To sum up, functionally, Yap1 is the major regulator of oxidative stress, Yap2 of cadmium stress, Yap4 and Yap6 of osmotic stress and Yap8 of arsenic stress [89] in *S. cerevisiae*. There is also evidence of cross-talk between Yap members. For instance, the *yap1yap2 *double mutant is more sensitive to oxidative stress than either single mutant alone, as the *yap1yap8 *double mutant is to arsenic stress [90].

After sequencing the *K. lactis *genome, the ORF KLLA0A01760g has been proposed as the *YAP1 *ortholog and KLLA0D14399g as the *YAP5/YAP7 *ortholog [105]. We have carried out an extensive study of the *K. lactis *genome looking for Yap homologues. Additional File 2 shows the identities found between the *S. cerevisiae *Yap family and several *K. lactis *ORFs using Bl2seq alignment  between related proteins from *K. lactis *and *S. cerevisiae*. The Skn7 transcriptional factor, related to oxidative stress response [106-108] has also a homologue in *K. lactis*, KLLA0A10219g.

*S. cerevisiae *Yap1 and Yap2 are closely related to KLLA0A01760g and might be derived from a common ancestor. In this regard, experimental data have shown that the *KlYAP1 *gene (KLLA0A01760g) is able to complement in *S. cerevisiae *both *yap1 *and *yap2 *mutations [109]. Besides, gene disruption experiments in *K. lactis *indicate that the *KlYAP1 *gene is involved in both the oxidative and cadmium response pathways [109].

**Figure 5:**
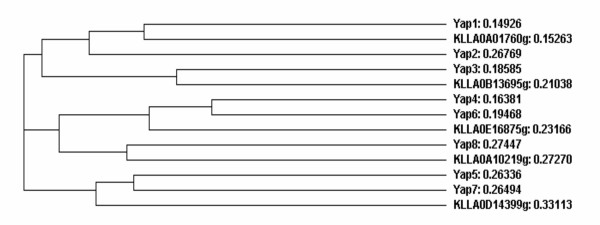
**Cladogram showing the phylogenetic relationships between the Yap factors from *S. cerevisiae *and *K. lactis *orthologs**. Distances are indicated after the protein or ORF names.

**Figure 6:**
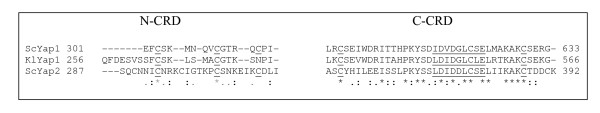
**Alignment of the N-CRD and C-CRD domains of *S. cerevisiae *Yap1, Yap2 and *K. lactis *Yap1**.

Data from Additional File 2 show that KLLA0B13695g is more closely related to Yap3, KLLA0E16875 to Yap4 and Yap6, KLLA0D14399g to Yap5 and Yap7 and KLLA0E00265g to Yap8. Using the alignments of the bZip domains, the cladogram (Figure 5) shows also the same relationship between *S. cerevisiae *and *K. lactis *genes. This suggests that, after genomic duplication [110,111]*Saccharomyces*-yeasts evolved by increasing the number of the Yap family of b-ZIP proteins. Figure 6 shows the conservation of the basic region and Leu zipper in the bZip domain of the *KlYAP *genes as well as positional coincidence of the domain in the topology of orthologs from *K. lactis *and *S. cerevisiae*. Experimental data on the function of these genes in *K. lactis *are necessary to clarify whether there is some functional specialization of these transcriptional factors related to specific forms of stress as previously reported in *S. cerevisiae*.

#### Yap1 and their partners in redox-sensing

In *S. cerevisiae*, Yap1 is activated upon exposure to oxidants by a mechanism which acts on its subcellular protein localization. In non-stressed cells, rapid nuclear export of Yap1 prevents its nuclear regulatory function. The Yap1 nuclear export signal (NES) is embedded in a Cys-rich domain located at the C-terminal part of the protein (C-CRD). There is another Cys-rich domain in Yap1, located at its N-terminus (N-CRD). Transitional redox conformation is converted into Yap1 due to the intramolecular reorganization of Cys disulphide bonds which cause NES or hidden exposition. Activation by increased levels of ROS requires both the C-CRD and N-CDR centres, while response to thiol reactive chemicals requires only C-CRD [50,112-118]. In *Kl*Yap1 there is a good conservation of Cys residues and the NES signal embedded in the C-CRD and two Cys from the N-CRD are also conserved (Figure 6). Therefore, the existence of mechanisms for redox-regulation of *Kl*Yap1 cellular location by ROS and thiol reactive chemicals is predictable. We have explored the *K. lactis *genome looking for orthologs of the necessary partners of Yap1 for this regulation, Orp1/Gpx3, Crm1 and Ybp1. Orp1 is a thiol peroxidase (Gpx3) that functions as a hydroperoxide receptor to sense intracellular hydroperoxide levels and transduces a redox signal to the Yap1 transcription factor [53]. Ycb1 is a protein required for oxidation of specific Cys residues of the transcription factor Yap1, resulting in the nuclear localization of Yap1 in response to stress [119]. Ybp2 has a significant role in resistance to oxidative stress and has a sequence similarity to Ybp1. KLLA0F06732g has 80% identity to Gpx3. KLLA0E16061g has 86% homology to Crm1. KLLA0C05698g has 50% homology to Ybp1 and 50% homology to Ybp2 but only one gene is present in *K. lactis*.

#### Other transcriptional factors related to oxidative stress in *K. lactis*

Besides the Yap family of transcription factors and their effectors discussed above, we have recently found that the transcriptional factor *Kl*Hap1, which in *S. cerevisiae *controls the activation of respiratory genes during aerobiosis and has unknown functions during anaerobiosis [39], in *K. lactis *it is involved in the oxidative stress response. Transcriptional expression of *KlHAP1 *is dependent on oxygen availability, increasing its expression in hypoxia. Deletion of *KlHAP1 *increases the resistance to oxidative stress or cadmium tolerance. Moreover, the induction of *KlYAP1 *and *KlTSA1 *after the addition of 0.5 mM H_2_O_2 _is repressed by *Kl*Hap1 [120]. This repressor effect of *Kl*Hap1 might be physiologically important in the context of a very active respiratory metabolism in *K. lactis*, prone to producing oxidative damage. The negative effect of *Kl*Hap1 on *KlYAP1 *and *KlTSA1 *expression would serve to attenuate this response. It has been reported that the *KlHAP1 *disruptant shows temperature-sensitive growth at low glucose concentration and that *Kl*Hap1 represses the expression of the major glucose transporter gene *RAG1 *[121]. The dual control of *Kl*Hap1 over the glucose transport, conditioning the respiro-fermentative metabolism of the cells, as well as over *KlYAP1 *and *KlTSA1 *is a new clue about the close interrelationship between control of metabolic fluxes and oxidative stress response.

### Biotechnological applications of redox-control in yeasts

Differential production of ROS or response to oxidative stress in yeast species or strains from the same species has not only a scientific interest but also biotechnological implications in several fields.

One of these fields is the use of yeasts as cell factories. *K. lactis *is one of the most important non-*Saccharomyces *yeasts used as a host for heterologous protein production [122]. In *K. lactis*, an increased amount of ROS is present in cells expressing high levels of heterologous proteins. This fact plays an important role in the limitation of recombinant protein production which has to be overcome by using engineered strains with increased ROS detoxification mechanisms, for example by overexpression of *KlSOD1 *[37].

*K. lactis *is able to metabolize the milk sugar lactose, for this reason the whey obtained as a by-product of cheese making is a suitable substrate for the culture of this yeast and heterologous protein production. The *K. lactis *transcriptome in synthetic and cheese whey media was compared by DNA-array analysis and it was found that several genes related to GSH metabolism and oxidative stress response are over-expressed in cheese whey; these include *KlGLR1 *(KLLA0E24112g), *KlGRX3 *(KLLA0C17842g), *KlCTA1 *(KLLA0D11660g), *KlSOD1 *(KLLA0E05522g), *KlGRX5 *(KLLA0B09636g), *KlCTT1 *(KLLA0D14685g) and *KlYHB1 *(KLLA0B14476g) encoding a nitric oxide oxidoreductase, a flavohemoglobin involved in nitric oxide detoxification that plays a role in oxidative and nitrosative stress responses. Moreover, the groups of genes of protein glycosilation and post-translational processing are also differentially expressed in the two media. These data give support to the reports on the benefits of using cheese whey and *K. lactis *for heterologous protein secretion [123].

Some yeast strains are used to ferment sugars into fuel ethanol or beverages. It has been shown that hypoxic fermentation in media containing high concentration of sugar causes stress conditions, which results in the production of ROS and triggers an antioxidant response, as well as in the fact that the ROS scavenging ability is involved in the maintenance of the fermentative ability of yeast strains used in industrial processes [124].

Glutathione has several uses in pharmacology, cosmetics and food industries, and companies are interested in producing it. Some yeast strains, such as *S. cerevisiae *and *Candida utilis*, are currently used for fermentative glutathione production on an industrial scale. Improved yields have been obtained by optimizing the culture media and conditions, through mutagenesis and by overexpression of the genes of glutathione biosynthesis, mainly *GSH1 *that catalyzes the limiting step [125-129]. In *K. lactis*, it has been described that GSH homeostasis is linked to the flocculation mechanism and a possible biochemical regulation of lectin expression by GSH levels in cells has been postulated [130]. This characteristic could be exploited in biotechnological processes, for example, some disinfection procedures use oxidants that influence GSH homeostasis and therefore the degree of microorganisms aggregation which, in its turn, might be involved in partial deficiency of such disinfection procedures [130].

Another field is the use of yeast mutants as models of aging research [131-133] and in human pathologies related to oxidative stress [134,135]. Although, once more, most studies have been performed with *S. cerevisiae*, the fermentative prototype, the differences found with *K. lactis *suggest the applicability of this respiratory yeast as an alternative model. For example, whereas in *S. cerevisiae *caloric restriction causes an increase in longevity, this does not occur in *K. lactis *[19]. Also, yeast cells that exclusively respire have been proposed as more reliable models of the highly oxidative neuronal metabolism [135]. Another example is the differential regulation of mitochondrial alternative dehydrogenases from the two yeasts [22]. Since these enzymes are not present in mammals, they are being used in the development of selective therapeutic drugs for pathogens [136]

## Conclusion

Whereas *S. cerevisiae *is a fermentative yeast considered the eukaryote model for studies on oxidative stress, *K. lactis *is a respirative yeast that emerges as an alternative model. The knowledge about the oxidative stress response pathways in *K. lactis *is hitherto little if compared with *S. cerevisiae *but the full genome sequences of both yeasts are available and studies based on sequence homology can be performed. This approach suggests that the same pathways of the oxidative stress response are present in both yeasts and that genes are generally conserved. However, several functional differences have appeared and they have been attributed to differences in their respiro-fermentative metabolism. These differences constitute new promising research fields and applied biotechnological implications are also envisaged.

## Competing interests

The authors declare that they have no competing interests.

## Authors' contributions

MEC and MIGS conceived of the study and participated in its design and coordination, performed the literature review and drafted the manuscript. Some results in references cited and unpublished, from the authors' laboratory, are part of the PhD works of NT and AGL. NT conceived and depicted Figure 1. AGL contributed to the construction of the models depicted in Figures 3 and 4. All authors read and approved the final manuscript.

## Supplementary Material

Additional file 1 **Alignment scores and predicted subcellular location for the putative oxidative stress response proteins in *K. lactis***. BLASTp results obtained for the putative oxidative stress response proteins in *K. lactis *and their *S. cerevisiae *counterparts (determined by compositional matrix adjustment), and subcellular location for the *K. lactis *proteins predicted by WoLF PSORT [36]. Click here for file

Additional file 2 **Alignment scores for the Yap family of b-ZIP proteins and Skn7 in S. cerevisiae and *K. lactis***. First and second lines: positives/overlap; third line: score (bits); fourth line: expect. Determined by compositional matrix adjustment
Click here for file
