# Supplementary material for: Sugar metabolism, redox balance and oxidative stress response in the respiratory yeast Kluyveromyces lactis
Source: Microb Cell Fact. 2009 Aug 30;8:46. doi: 10.1186/1475-2859-8-46 (PMC2754438; doi:10.1186/1475-2859-8-46)
Supplement: Additional file 1 — Alignment scores and predicted subcellular location for the putative oxidative stress response proteins in K. lactis. BLASTp results obtained for the putative oxidative stress response proteins in K. lactis and their S. cerevisiae counterparts (determined by compositional matrix adjustment), and subcellular location for the K. lactis proteins predicted by WoLF PSORT [36]. [file 1475-2859-8-46-S1.pdf]

Alignment scores and predicted subcellular location for the putative oxidative stress response proteins in *K. lactis*.

BLASTp results obtained for the putative oxidative stress response proteins in *K. lactis* and their *S. cerevisiae* counterparts (determined by compositional matrix adjustment), and subcellular location for the *K. lactis* proteins, predicted by WoLF PSORT [36].

**Gene**	**ORF *K. lactis***	**ORF *S.cerevisiae***	**BLASTp pairwise**	**Predicted subcellular location of *K. lactis* protein**
**Chaperones for superoxide dismutases**				
CCS1	KLLA0F26917g 245 Aa	YMR038c 249 Aa	Sc = 278 bits (712), Ex = 1e-79, Id = 135/248 (54%), Pos = 185/248 (74%), G = 7/248 (2%)	cyto_nucl: 13.5, cyto: 13.0, nucl: 10.0
MTM1	KLLA0A09383g 366 Aa	YGR257c 366 Aa	Sc = 432 bits (1111), Ex = 1e-125, Id = 218/367 (59%), Pos = 267/367 (72%), G = 15/367 (4%)	nucl: 8.0, mito: 6.0, plas: 6.0, cyto: 5.0
**Superoxide dismutases**				
SOD1	KLLA0E05567g 155 Aa	YJR104C 154 Aa	Sc = 229 bits (585), Ex = 1e-65, Id = 108/154 (70%), Pos = 124/154 (80%), G = 0/154 (0%)	cyto: 18.5, cyto_nucl: 13.5, extr: 4.0, nucl: 3.5
SOD2	KLLA0E03609g 226 Aa	YHR008C 233 Aa	Sc = 353 bits (906), Ex = 2e-102, Id = 169/232 (72%), Pos = 196/232 (84%), G = 8/232 (3%)	mito: 20.5, mito_nucl: 11.8, cyto: 4.5, cyto_nucl: 3.8
**Catalases**				
CTA1	KLLA0D11660g 511 Aa	YDR256c 515 Aa	Sc = 867 bits (2241), Ex = 0.0, Id = 396/492 (80%), Pos = 441/492 (89%), G = 1/492 (0%)	pero: 10.0, cyto: 9.0, cyto_nucl: 8.5, nucl: 6.0
CTT1	KLLA0D14685g 550 Aa	YGR088w 562 Aa	Sc = 813 bits (2099), Ex = 0.0, Id = 377/551 (68%), Pos = 448/551 (81%), G = 14/551 (2%)	nucl: 11.0, cyto_nucl: 10.5, cyto: 8.0, mito: 4.0, pero: 4.0
**Glutathione peroxidases**				
GPX2	KLLA0D03905g 168 Aa	YBR244w 162 Aa	Sc = 197 bits (502), Ex = 6e-56, Id = 92/160 (57%), Pos = 118/160 (73%), G = 0/160 (0%)	cyto: 17.5, cyto_nucl: 11.5, nucl: 4.5, mito: 3.0
GPX3	KLLA0F06732g 161 Aa	YIR037w 163 Aa	Sc = 275 bits (702), Ex = 3e-79, Id = 129/161 (80%), Pos = 148/161 (91%), G = 0/161 (0%)	cyto: 17.5, cyto_nucl: 13.8, nucl: 7.0, mito_nucl: 5.5
**Thioredoxin peroxidases**				
TSA1	KLLA0B01628g 197 Aa	YML028w 196 Aa	Sc = 365 bits (936), Ex = 4e-106, Id = 174/196 (88%), Pos = 186/196 (94%), G = 0/196 (0%)	cyto: 13.0, cyto_nucl: 8.5, E.R.: 6.0, extr: 3.0, nucl: 2.0
AHP1	KLLA0F20009g 171 Aa	YLR109w 176 Aa	Sc = 171 bits (432), Ex = 8e-48, Id = 83/162 (51%), Pos = 111/162 (68%), G = 4/162 (2%)	cyto: 13.5, cyto_nucl: 10.0, mito: 8.0, nucl: 3.5
AHP1	KLLA0A07271g 181 Aa	YLR109w 176 Aa	Sc = 84.3 bits (207), Ex = 3e-17, Id = 58/145 (40%), Pos = 83/145 (57%), G = 9/145 (6%)	cyto: 16.0, mito: 11.0
PRX1	KLLA0E20285g 249 Aa	YBL064C 261 Aa	Sc = 343 bits (881), Ex = 1e-99, Id = 161/242 (66%), Pos = 197/242 (81%), G = 3/242 (1%)	mito: 17.0, nucl: 7.5, cyto_nucl: 5.0

PRX1	KLLA0A02651g<br>224 Aa	YBL064C<br>261 Aa	Sc = 190 bits (482), Ex = 2e-53,<br>Id = 95/212 (44%), Pos = 134/212 (63%), G = 7/212 (3%)	mito: 11.0, nucl: 8.0, cyto_nucl: 7.0,<br>cyto: 4.0, pero: 4.0
DOT5	KLLA0D14333g<br>205 Aa	YIL010W<br>215 Aa	Sc = 236 bits (603), Ex = 3e-67,<br>Id = 119/205 (58%), Pos = 156/205 (76%), G = 6/205 (2%)	nucl: 15.0, cyto_nucl: 11.8,<br>mito_nucl: 10.0, cyto: 5.5, mito: 3.5
**Glutathione synthesis**				
GSH1	KLLA0F14058g<br>665 Aa	YJL101C<br>678 Aa	Sc = 892 bits (2306), Ex = 0.0,<br>Id = 423/673 (62%), Pos = 529/673 (78%), G = 14/673 (2%)	nucl: 10.5, cyto_nucl: 10.5, cyto: 7.5,<br>pero: 5.0, mito: 3.0
GSH2	KLLA0F07557g<br>487 Aa	YOL049w<br>491 Aa	Sc = 528 bits (1359), Ex = 5e-154,<br>Id = 262/479 (54%), Pos = 350/479 (73%), G = 14/479 (2%)	nucl: 17.5, cyto_nucl: 13.5, cyto: 6.5
**Thioredoxins**				
TRX1	KLLA0E16347g<br>104 Aa	YLR043C<br>103 Aa	Sc = 148 bits (374), Ex = 2e-41,<br>Id = 70/103 (67%), Pos = 88/103 (85%), G = 1/103 (0%)	cyto: 14.0, cyto_nucl: 10.5, nucl: 5.0,<br>mito: 5.0
TRX3	KLLA0F10351g<br>139 Aa	YCR083w<br>127 Aa	Sc = 127 bits (318), Ex = 7e-35,<br>Id = 59/120 (49%), Pos = 84/120 (70%), G = 4/120 (3%)	mito: 19.5, cyto_mito: 13.8, cyto: 7.0
**Thioredoxin reductase**				
TRR1	KLLA0E21605g<br>349 Aa	YDR353W<br>319 Aa	Sc = 596 bits (1537), Ex = 2e-175,<br>Id = 281/319 (88%), Pos = 301/319 (94%), G = 0/319 (0%)	mito: 20.5, cyto_mito: 12.5, cyto: 3.5
TRR1	KLLA0F15037g<br>297 Aa	YDR353W<br>319 Aa	Sc = 41.6 bits (96), Ex = 2e-08,<br>Id = 48/180 (26%), Pos = 72/180 (40%), G = 16/180 (8%)	cyto: 14.0, cyto_nucl: 9.0, cysk: 9.0,<br>nucl: 2.0
**Glutaredoxins**				
GRX3	KLLA0C17842g<br>264 Aa	YDR098c<br>285 Aa	Sc = 323 bits (828), Ex = 5e-93,<br>Id = 169/267 (63%), Pos = 199/267 (74%), G = 20/267 (7%)	cyto_nucl: 14.8, cyto: 12.0, nucl: 11.5,<br>mito_nucl: 7.5
GRX5	KLLA0B09636g<br>142 Aa	YPL059w<br>150 Aa	Sc = 210 bits (534), Ex = 1e-59,<br>Id = 99/133 (74%), Pos = 114/133 (85%), G = 0/133 (0%)	mito: 22.5, cyto_mito: 13.5, cyto: 3.5
GRX6	KLLA0E17733g<br>211 Aa	YDL010w<br>231 Aa	Sc = 133 bits (334), Ex = 8e-36,<br>Id = 69/162 (42%), Pos = 102/162 (62%), G = 10/162 (6%)	extr: 14.0, E.R.: 4.0, golg: 4.0, mito: 3.0
GRX8	KLLA0B07975g<br>111 Aa	YLR364w<br>109 Aa	Sc = 137 bits (344), Ex = 5e-38,<br>Id = 64/110 (58%), Pos = 80/110 (72%), G = 2/110 (1%)	cyto: 11.5, cyto_mito: 10.7,<br>cyto_nucl: 9.3, mito: 8.5, nucl: 5.0
**Glutathione reductase**				
GLR1	KLLA0E24112g<br>484 Aa	YPL091w<br>483 Aa	Sc = 747 bits (1929), Ex = 0.0,<br>Id = 353/470 (75%), Pos = 417/470 (88%), G = 2/470 (0%)	mito: 11.0, cyto_nucl: 7.5, nucl: 7.0,<br>cyto: 6.0
**Glutathione transferases**				
GTT1	KLLA0A00264g<br>234 Aa	YIR038c<br>234 Aa	Sc = 302 bits (774), Ex = 3e-87,<br>Id = 151/234 (64%), Pos = 191/234 (81%), G = 0/234 (0%)	mito: 18.0, nucl: 5.5, cyto_nucl: 5.0,<br>cyto: 3.5
GTO2	KLLA0F12056g<br>361 Aa	YKR076w<br>370 Aa	Sc = 491 bits (1263), Ex = 4e-143,<br>Id = 241/370 (65%), Pos = 296/370 (80%), G = 9/370 (2%)	nucl: 11.0, cyto_nucl: 9.0, mito: 7.0,<br>cyto: 5.0, pero: 2.0

Abreviations: cyto (cytosol), cysk (cytoskeleton), E.R. (endoplasmic reticulum), extr (extracellular), golg (Golgi apparatus), lyso (lysosome), mito (mitochondria), nucl (nuclear), pero (peroxisome), plas (plasma membrane), vacu (vacuolar membrane). Sc (score), Ex (expect), Id (identities), Pos (Positives), G (gaps)
