# Supplementary material for: Sugar metabolism, redox balance and oxidative stress response in the respiratory yeast Kluyveromyces lactis
Source: Microb Cell Fact. 2009 Aug 30;8:46. doi: 10.1186/1475-2859-8-46 (PMC2754438; doi:10.1186/1475-2859-8-46)
Supplement: Additional file 2 — Alignment scores for the Yap family of b-ZIP proteins and Skn7 in S. cerevisiae and K. lactis. First and second lines: positives/overlap; third line: score (bits); fourth line: expect. Determined by compositional matrix adjustment [file 1475-2859-8-46-S2.pdf]

Alignment scores for the Yap family of b-ZIP proteins and Skn7 in *S. cerevisiae* and *K. lactis.*

S. cerevisiae / *K. lactis*	Yap1 (651 Aa)	Yap2 (409 Aa)	Yap3 (330 Aa)	Yap4 (295 Aa)	Yap5 (245 Aa)	Yap6 (383 Aa)	Yap7 (245 Aa)	Yap8 (294 Aa)	Skn7 (622 Aa)
KLLA0A01760g (583 Aa)	76/111 (68%) 118 6e-31	162/336 (48%) 80.9 9e-20	55/92 (59%) 51.6 5e-11	37/57 (64%) 47.4 9e-10	43/74 (58%) 39.7 2e-07	22/27 (81%) 40.0 2e-07	41/69 (59%) 42.0 2e-08	48/77 (62%) 41.6 4e-08	
KLLA0B13695g (375 Aa)	51/79 (64%) 48.1 6e-10	53/83 (63%) 40.0 1e-07	116/191 (60%) 135 2e-36	45/87 (51%) 37.0 8e-07	36/57 (63%) 2.7 1e-05	35/60 (58%) 38.5 4e-07	28/40 (70%), 32.3 2e-05	19/41 (46%) 22.3 0.017	
KLLA0E16875g (508 Aa)	32/52 (61%) 45.4 6e-09	20/27 (74%) 36.6 2e-06	35/59 (59%) 39.7 2e-07	82/130 (63%) 91.3 4e-23	37/63 (58%) 35.8 2e-06	48/62 (77%) 73.6 1e-17	26/47 (55%) 35.8 2e-06	10/13 (76%) 21.6 0.038	
KLLA0D14399g (525 Aa)	26/41 (63%) 38.9 6e-07	22/34 (64%) 35.8 4e-06	21/28 75%) 34.7 6e-06	19/31 (61%) 28.1 4e-04	79/164 (48%) 51.2 4e-11	51/114 (44%) 38.1 5e-07	75/160 (46%) 57.8 4e-13	19/48 (39%) 20.8 0.075	
KLLA0E00265g (288 Aa)	25/34 (73%) 38.5 4e-07	34/52 (65%) 39.3 1e-07	42/82 (51%) 40.0 7e-08	31/53 (58%) 37.4 4e-07	44/82 (53%) 31.6 2e-05	24/37 (64%) 31.2 3e-05	35/71 (49%) 37.0 5e-07	141/319 (44%) 82.4 1e-20	
KLLA0A10219g (475 Aa)									273/419 (65%) 382 1e-110

First and second lines: positives/overlap; third line: score (bits); fourth line: expect. Determined by compositional matrix adjustment
